# Pushing the boundary of child well-being: A spatial examination of child mortality in transition zones of extreme economic inequality and material hardship

**DOI:** 10.1371/journal.pone.0330449

**Published:** 2025-08-25

**Authors:** Gia Elise Barboza-Salerno, Sharefa Duhaney, Brittany Liebhard, Karla Schockley-McCarthy

**Affiliations:** 1 College of Public Health, The Ohio State University, Columbus, Ohio, United States of America; 2 College of Social Work, The Ohio State University, Columbus, Ohio, United States of America; 3 College of Medicine, The Ohio State University, Columbus, Ohio, United States of America; Independent, UNITED STATES OF AMERICA

## Abstract

How do patterns of socioeconomic inequality shape the risk of child fatality in urban areas? Research has shown that both intentional and unintentional child deaths are concentrated in areas of social disadvantage. Yet in densely populated cities, child fatality risk may not follow smooth spatial patterns and instead exhibit abrupt shifts across neighborhood boundaries. This study applies a dissimilarity-based Bayesian spatial conditional autoregressive (CAR) model to detect localized discontinuities in child mortality risk associated with structural inequality. Using a continuous dissimilarity function based on differences in Economic Hardship and Inequality (EHI) scores between adjacent census tracts, the model allows spatial smoothing to attenuate across sharp transitions. The model identified 413 spatial boundaries—termed social frontiers—where sharp structural discontinuities in EHI were associated with a 22% increase in the relative risk of child mortality, even after adjusting for racial segregation, concentrated disadvantage, residential mobility, and immigrant concentration. The significance of identifying neighborhoods characterized as social frontiers, where children may benefit from additional preventive interventions, is discussed in this context.

## Introduction

Child mortality is a critical public health issue in the United States. In 2021, the all-cause child mortality rate reached its highest level since 2008, with significant increases observed during the COVID-19 pandemic [1, 2]. In Cook County, Chicago, the geographic location of the present study, 599 children under the age of 18 died in 2022, a 15.9% increase from the previous year [3, 4]. These figures mask substantial heterogeneity in child mortality across race, socioeconomic status, and geography. More than half (56.6%) of all deaths of children occurred in the city of Chicago. Cook County exemplifies the national trend of Black children’s disproportionate mortality rates, with non-Hispanic Black children making up 61.5% (*N = *1,369) of all child fatalities between 2015 and 2023, while comprising only 23.6% of the child population. On the other hand, non-Hispanic White children were significantly underrepresented compared to their share of the population, comprising 16.2% of the deaths but 65.1% of children [5].

Significant disparities in child mortality rates across regions and socioeconomic groups indicate that economic inequality perpetuates existing racial and ethnic disparities. Geographic location is a key factor in child mortality, influencing access to healthcare, the quality of public health infrastructure, and the extent of socioeconomic challenges. Urban areas with high levels of poverty are particularly vulnerable, with children facing increased risks of mortality due to inadequate housing, environmental hazards, and limited access to quality healthcare. Low-income families are disproportionately affected, as children in these households often experience poor health outcomes driven by factors such as insufficient nutrition [6–8], limited access to healthcare [9–11], and greater exposure to environmental toxins [12, 13]. These conditions contribute to a higher prevalence of adverse health outcomes, including preterm births, low birth weight, and chronic illnesses, all of which significantly raise the risk of infant and child mortality [14].

The existing literature on child mortality disparities across income levels primarily focuses on individuals or households, often neglecting the role of neighborhood-level economic inequality [15, 16]. While it is well-documented that child mortality rates vary geographically and are influenced by socioeconomic factors, no study to date has directly examined how economic inequality between neighborhoods relates to these spatial disparities in child deaths [17]. This study addresses that gap by investigating whether area-level income gradients—particularly sharp socioeconomic differences between adjacent neighborhoods—influence the relative risk of child death. Our analysis captures the impact of spatial economic inequalities that are not fully explained by conventional measures of social vulnerability.

### Economic inequality and child mortality

Despite significant declines in child mortality rates since 1950, socio-economic disparities in child mortality across levels of socio-economic disadvantage have not only persisted [18], but they have increased [19]. During the past four decades, income inequality in the Chicago region, for example, increased by nearly 36%, surpassing the 19% rate of increase for the United States [20]. Socioeconomic inequality is a reflection of housing insecurity, lack of job opportunities, quality education, healthcare, and childcare, and remains a critical social determinant of mortality among youth [16,19]. Spatial concentrations of child mortality risk have been attributed to variations in material wealth across communities [21]. Past research has demonstrated socioeconomic gradients in all-cause child mortality and specific-cause mortality in vehicular accidents [22], pedestrian injury [23], fires [24, 25], drowning [26], sudden infant death syndrome [27], child maltreatment [28], and congenital conditions [29]. The survival advantage of children who live in the least deprived neighborhoods has been attributed to the differences in quality education, employment, and healthcare opportunities found in wealthier areas [18,30]. Not all socio-economically disadvantaged neighborhoods within the same geographic region experience the same level of inequality, however. In metropolitan areas, the observed spatial heterogeneity in child mortality outcomes is influenced by a diverse range of social, economic, and environmental determinants that vary significantly depending on the region being considered [19,31–33]^.^ Neighborhood structural disadvantage has been reinforced by punitive justice-related and discriminatory housing policies, as well as antipoverty policies that aim to supervise and adjudicate family life in impoverished areas, serving as pathways by which structural determinants shape health outcomes for children [34, 35].

### Neighborhood boundaries as social frontiers

Neighborhood boundaries may be defined as homogenous areas, such as census tracts or counties, or as spatial zones of rapid change often referred to as *social frontiers* [35–37]. Even when neighborhoods exhibit internal similarity based on specified characteristics, they may differ significantly in terms of their sociospatial positioning, as measured by how they compare to adjacent areas. Social frontiers, which represent spatial divisions in racial, ethnic, religious, cultural, or social characteristics that separate neighboring communities, act as “cliff edges” between adjacent neighborhoods [35]. These boundaries are scientifically important because they mark areas where social or economic conditions shift abruptly, distinguishing characteristics on one side from those on the other [37]. Unlike conventional boundaries, social frontiers are open, meaning they appear as line segments (i.e., not polygons) and do not enclose discrete areas. Because the processes that give rise to boundaries are not always associated with homogeneous areas, social frontiers may matter more on “one side where they divide Black [*sic*] city residents from surrounding White [*sic*] suburban areas, but they may not matter on another side (p. 20)” [38].

Social frontiers typically coincide with the edges of highly segregated regions where neighboring areas are more homogenous [38–41]. Social frontiers may be caused by a “strong aversion [among residents] to living at the interface of communities in conflict (pg. 274)” [35,42] or because of “benign forces” [35,43] and unintended consequences of micro-level (i.e., individual) decisions. Alternatively, social frontiers may emerge from explicit and intentional policies that have prevented people in lower-income communities of color from accumulating wealth and investing in home ownership rather than being an “accident of economic circumstance, demographic trends, personal preference, and private discrimination” [44]. Regardless of their origins, social frontiers are places of stark spatial divisions with complex social consequences. Such variation has important implications for understanding the impact of socio-spatial segregation and its disparate effects on health and well-being. These boundaries demarcate areas with differences in level of service and resource distribution, cultural and place-based identity, and social interactions, all of which can impact the social dynamics of these areas [45]. Residents’ social interactions and demographic characteristics shape the meaning and perception of the places where people live, even within the same geographic area [46].

### How do social frontiers affect child mortality risk?

To fully understand the impact of spatial opportunity structure on health and well-being, it is essential to look beyond the characteristics and interactions within individual neighborhoods [45,47]. More specifically, it is essential also to consider spillover effects—how conditions in one neighborhood impact the health and well-being of neighboring areas [45]. Externality space theory provides an organizing framework for conceptualizing differences across bordering areas. According to the theory, places “where individuals do not share externalities with proximate others is axiomatically a boundary between … neighborhoods … that identify schisms in the social meaning or attributes of space at a given point … (pg. 83).” Boundary areas significantly alter the benefits and detriments that individuals derive from a particular location [45].

Externality space theory identifies three core dimensions that help explain how and why boundaries shape neighborhood-level outcomes: congruence, generality, and accordance. *Congruence* refers to the level of concordance between the externality space and a defined geographic boundary. In the present case, the congruence between EHI and child mortality risk is high when confined to specific neighborhoods. The *generality* of an externality space refers to how consistently an individual’s well-being is mapped to the same area. As applied here, if externalities related to neighborhood economic inequality (e.g., healthcare access) are associated with increased child mortality risk, then the externality space has high generality. *Accordance* refers to whether all individuals share a common understanding about where the boundaries of EHI begin and end (i.e., the externality space). The externality space theory of neighborhood boundaries introduces a novel approach to understanding urban dynamics [45] because it emphasizes important linkages across different sociospatial contexts— including the interrelatedness between space, sociability, and spatial conditions [34]. In short, this perspective highlights residents’ social marginalization due to the imposed nature of neighborhood physical boundaries.

At social frontiers, spatial proximity may decrease social interaction and the sharing of social institutions across groups [48]. Social frontiers may erect barriers to increased social interactions between neighbors, leading to greater mistrust and misunderstanding between groups, lower levels of collective efficacy, and increased child mortality [35,49]. This is supported by Legewie (2018), who found that even after considering traditional indicators of social disorganization, including residential instability, immigrant concentration, ethnic heterogeneity, and concentrated disadvantage, racial neighborhood boundaries continued to predict violent crime and homicide risk in Chicago. According to Legewie, boundary areas create opportunities for criminal behavior due to the lack of social control and cohesion that characterizes adjacent homogeneous regions, as well as the potential for intergroup conflict. Legewie (2018) used a technique known as ‘areal wombling’ to detect boundaries based on sharp changes in racial-ethnic composition. Drawing on multiple theories, he claims that “...boundaries lack the social control and cohesion of adjacent homogeneous areas; are contested between groups, provoking intergroup conflict; and create opportunities for criminal behavior” (Legewie, 2018, p 1958). In this study, violent crime was higher at boundary locations, representing sharp delineations in racial-ethnic composition, net of neighborhood characteristics, measures of spatial interdependence, and other physical boundaries. Kim and Hipp (2018) similarly found that proximity to the city boundary, as a distance decay, predicted increased motor vehicle theft [50]. Boundaries between two areas have been shown to increase crime opportunities, partly because of the possibility of fewer guardians, or the transitory nature of these locations, making escape quicker and easier for perpetrators of violent crime [51].

Regarding child mortality specifically, the perception of relative resource deprivation can lead to social disenfranchisement, mental distress, and poor health outcomes, particularly if the deprivation is attributed to social stigma and racism [48]. Research shows that the overlapping and reinforcing effects of residential segregation are associated with higher rates of infant mortality and preterm birth among Black infants [52,53]. One study found that the excess risk of mortality among preterm infants born in neighborhoods characterized by high relative concentrations of Black and poor residents is explained in part by the hospital in which they receive care [52]. More specifically, infants from predominantly Black neighborhoods experienced worse outcomes when born in hospitals located in similarly disadvantaged areas than when born in hospitals located in neighborhoods with a higher proportion of White residents.

In contradistinction, studies have shown that spatial proximity can increase, rather than decrease, social interaction and the sharing of institutional resources across regions [48]. For example, ethnic diversity and multiculturalism have been viewed as positive externalities because they promote adherence to positive health behaviors consistent with child well-being through disease prevention and information about healthcare service locations [54–57]. Cross-cultural work has demonstrated locational advantages associated with proximity to affluent neighbors, such as greater accessibility to labor market opportunities and economic integration, but only when public spaces are shared effectively by socially dissimilar groups [33]. Moreover, increased social interaction in heterogeneous spaces can mitigate the impact of negative stereotypes and group threats and increase trust across groups [58,59]. In the context of child mortality, behavioral changes resulting from social interaction across diverse environments may be crucial for improving children’s survival. For instance, one study found that infant mortality is lower among rural-urban migrants than rural non-migrants, highlighting the role of social interaction in exposing individuals to new ideas, which can lead to changes in attitudes, lifestyles, and motivations [60].

### Current study

Past research confirms the importance of neighborhood boundaries as defining aspects of highly segregated urban areas; however, the precise nature of this association remains challenging to predict. Living in proximity to a social frontier may impact child mortality by increasing access to buffering resources that can mitigate poor child health outcomes for those living in highly disadvantaged areas [61]. On the other hand, living near a social frontier may lead to social isolation and lower levels of collective efficacy, which can result in an increased mortality risk. To fully examine the effect of spatial inequality on child mortality, it is critical to consider multiple measures of economic inequality across and within regions. Boundary analysis captures the transition from low to high-class neighborhoods by detecting statistically significant latent discontinuities over a geographic surface [49,62]. In the present study, a novel form of spatial analysis called boundary detection is used to detect social frontiers of EHI – neighborhood boundaries based on extreme differences in economic inequality – and their association with child fatality in Cook County, Chicago. We hypothesized that living near a social frontier would be associated with decreased mortality due to the potential flow of resources from areas of less disadvantage.

## Methods

### Data

We extracted all-cause mortality data for children under 18 from 2015–2023 from the Cook County Medical Examiner’s Office’s (CCMEO) publicly available database [63]. The CCMEO investigates deaths due to criminal violence, accidents, suicides, and other suspicious or unusual circumstances. The data include detailed records of deaths occurring in Cook County, Illinois, from August 2014 to the present and are updated daily. Records include cause and manner of death, demographic characteristics, and other relevant information regarding the death.

To measure neighborhood-level socioeconomic conditions, we used the Economic Hardship and Inequality (EHI) Index, a tract-level subcomponent of the Area Deprivation Index (ADI) [64]. The ADI is a composite measure of disadvantage derived from the American Community Survey (ACS) 5-year estimates (2015–2019) [65].

## Variables

### Dependent variable

***Child Fatalities***. Observed counts associated with each child death location, $Y=\;\left(Y_{1},\ldots Y_{K}\right),$ were aggregated within census tracts. The number of children under 18 in each census tract in the county was downloaded from the American Community Survey (ACS) based on 5-year estimates for 2011–2015 and 2015–2019. Based on population size, the expected child fatality count, $E=\;\left(E_{1},\ldots E_{K}\right),$ for each census tract was computed using the *SpatialEpi* [66] package in R.

### Independent variable

***Economic Hardship and Inequality (EHI) Index*** The ADI is a composite measure of disadvantage derived from the American Community Survey (ACS) 5-year estimates (2015–2019), incorporating indicators related to income, education, employment, and housing quality. The EHI subindex specifically reflects spatial patterns of economic deprivation, integrating measures such as the ratio of households earning under $10,000 to those earning over $50,000, the percentage of owner-occupied housing, unemployment rates, and vehicle access. EHI scores are standardized to facilitate within- and between-state comparisons. We downloaded tract-level EHI data using the *get_adi* function from the *sociome* package in R (version 4.3.2). For additional detail on scale construction, see [24,64,67].

***Covariates*** Our models used multiple measures of social disorganization by creating indices corresponding to concentrated disadvantage, residential instability, immigrant concentration, and residential segregation similar to past research [49,68–70]. Following Legewie [49]: (1) concentrated disadvantage is a five-item index (eigenvalue 2.962; 59.2% variance) comprised of the following measures: poverty rate (factor loading: 0.847), unemployment rate (0.832), percentage of professional and management jobs (−0.681), the share of high-school graduates (0.616), and share single mother families (0.842); (2) residential instability is a three-item index (eigenvalue 1.85, 61.5% variance) comprised of the percentage of renter-occupied units (0.998), share of residents who moved to another dwelling since 2005 (0.490), and housing unit rental vacancy rate (0.781); (3) immigrant concentration is a three-item index (eigenvalue 2.54, 84.7% variance) comprised of the share of foreign-born residents (0.913), the share of residents who speak English less than “very well” (0.998), and the share of Spanish-speaking residents (0.844). Notably, the positive loading on the variable measuring educational attainment on the factor we labeled concentrated disadvantage may appear counterintuitive at first. However, this variable is defined as the number of individuals whose highest level of educational attainment is a high school diploma or equivalent. In this context, a higher proportion reflects limited access to higher education, which aligns with greater socioeconomic disadvantages. To measure the level of racial or ethnic segregation, or the concentration of different racial or ethnic groups within each neighborhood, we computed the Hirschman-Herfindahl Index (HHI). The HHI is calculated by squaring the proportion of the population that belongs to each racial or ethnic group and then summing the squared values. This index ranges from 0 to 1, where a value closer to 0 indicates complete diversity (i.e., a more even distribution of racial or ethnic groups), and a value closer to 1 indicates a highly segregated neighborhood. All social disorganization variables were derived from the American Community Survey (ACS) 5-year estimates for 2015–2019 [65].

### Statistical analysis.

**Fig 1:**
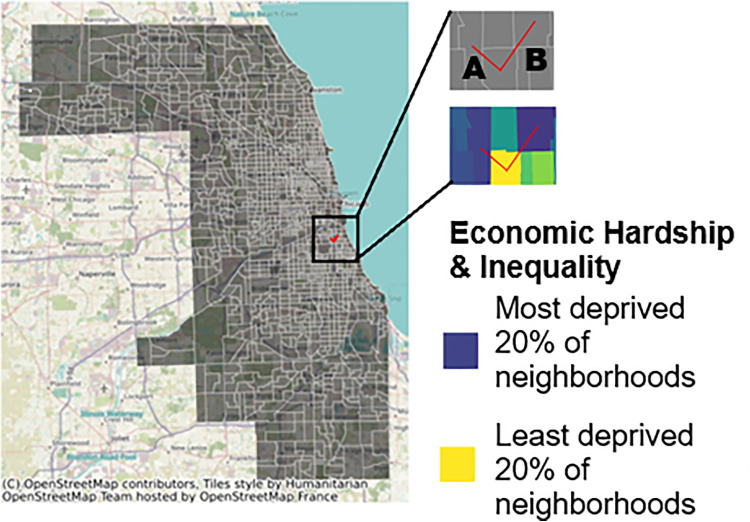
Illustration of the boundary detection method. The figure shows a census tract that neighbors two other census tracts labeled by lines A and B (left). The figure to the right shows that this tract was categorized as one of the least deprived 20% of neighborhoods by the EHI index, as shown in yellow. However, it borders two census tracts classified as the most deprived 20% of census tracts by the EHI.

***Overview of boundary detection approach.*** Our analysis proceeded as follows. We first calculated the decile-based EHI score for each census tract and computed the absolute difference in EHI deciles between geographically adjacent areas. Geographic adjacency was defined using a binary spatial weights matrix *W*, constructed from first-order contiguity via the *poly2nb* and *nb2mat* functions in R. We then generated a normalized dissimilarity matrix *Z*, where each element z_*ij*_ represents the scaled absolute difference in EHI deciles between adjacent census tracts *i* and *j,* such that $z_{ij}=\frac{\left|{EHI}_{i}-{EHI}_{j}\right|}{max(\left|{EHI}_{i}-{EHI}_{j}\right|)}.$ We examined the distribution of EHI differences between neighboring areas to identify geographic borders where these differences are statistically significant. To model the relationship between child deaths and neighborhood disadvantage, we employed a dissimilarity-based conditional autoregressive model proposed by Lee [70] and Lee & Mitchel [71]. This approach incorporates spatial smoothing while allowing for spatial discontinuities across structural boundaries. The modified spatial neighborhood matrix, W*, is calculated as $w_{ij}^{*}=w_{ij}\times exp({-z}_{ij})$ where $w_{ij}$ is 1 if tracts *i* and *j* are adjacent and 0 otherwise. This specification ensures that spatial dependence is preserved among similar neighbors and gradually attenuated across areas with greater EHI dissimilarity, rather than enforcing an arbitrary threshold. All contiguous pairs contributed to the spatial structure in the final model, with greater smoothing across socioeconomically similar tracts and reduced smoothing across potential “social frontiers.” To visualize high-contrast boundaries, differences in EHI across adjacent tracts were visually mapped to show areas of greater structural inequality. Fig 1 illustrates a region characterized by a significant difference in EHI across boundaries.

***Modeling*** Following Lee and Mitchell (2012), a Bayesian spatial CAR model was specified as


$$Y_{k}|E_{k},R_{k}\sim Poisson\left(E_{k}R_{k}\right)\;for\;k=1,\ldots ,K$$ (1)

$$\text{ln}(R_{k})=x_{k}^{T}\beta +\phi_{k},$$ (2)

where the risk of mortality in area *k* is denoted by $R_{k}$, $x_{k}^{T}$is the matrix of covariates representing indicators of social disorganization, and the random effects are specified as $\phi \;=\left({\phi \;}_{1},\ldots ,{\phi \;}_{K}\right).\;$The random effects allow for overdispersion and spatial correlation in the data, even after controlling for the effects of social disorganization. Following Lee [71] and Lee and Mitchell [72] the S.CARdissimilarity function in R was used to run the localized CAR model, including the *K x K* dissimilarity matrix. As applied to the present analysis, incorporating the matrix into the modeling scheme allowed us to identify the magnitude of the difference in EHI between two areas. It was used to inform the spatial adjacency matrix governing the random effects structure, rather than being entered as a fixed-effect predictor. In this model, the random effects in neighborhoods that share a border are modeled as correlated or conditionally independent, depending on whether EHI in the two bordering areas is similar (correlated random effects) or very different (conditionally independent) [73]. Spatial correlation is induced using a CAR prior for the random effects via the *K x K* neighborhood matrix W*. We fit three separate boundary detection models: (1) a model without covariates; (2) a model with concentrated disadvantage, residential mobility, racial/ethnic diversity, and immigrant concentration; and (3) a final model that included spatially lagged versions of each covariate. Spatially lagged variables were calculated as spatially weighted averages of each variable across neighboring census tracts, using a predefined spatial weights matrix. We evaluated the presence of multicollinearity using Variance Inflation Factors (VIFs). In the base model without lags, all VIFs were well below 5. After adding lags, values increased moderately (e.g., concentrated disadvantage lag = 6.10; immigrant concentration lag = 5.00), but remained within an interpretable range and were lower than those reported by Legewie (2018) in a similar analysis. To further assess collinearity, we examined posterior correlations among regression coefficients. Strong negative correlations were observed between the variable concentrated disadvantage and its lag = –0.70, suggesting that these effects should be interpreted jointly rather than independently.

Inference for all models was based on 10,000 post-burn-in and thinned Markov Chain Monte Carlo (MCMC) samples and model convergence was based on Geweke diagnostics [74,75]. The residuals were assessed for spatial correlation using Moran’s I permutation test. Moran’s I measures the degree of spatial autocorrelation, indicating whether similar values are clustered or dispersed across a geographic area. A permutation test is used to assess whether the observed Moran’s I value is statistically significant, comparing it to a distribution of Moran’s I values generated under random spatial arrangements [76,77].

This is a retrospective study using publicly available archived records, and all data were fully anonymized before being accessed on March 26, 2024. According to the U.S. Department of Health and Human Services Policy for Protection of Human Research Subjects, research involving deceased persons is not considered human subjects research. Therefore, the study was deemed exempt from Institutional Review Board review.

## Results

Between 2015 and 2023, the Cook County Medical Examiner investigated 2,226 deaths of children under 18. Table 1 presents the descriptive characteristics of the most (and least) deprived 10% of neighborhoods. By definition, the most deprived 10% of neighborhoods exhibited extreme levels of EHI compared to the least deprived 20% (most deprived: mean = 142, SD = 10; least deprived: mean = 74.4, SD = 2). Notably, the least deprived 10% of neighborhoods had less variability in EHI scores. On average, there were 1.59 child deaths in the most deprived neighborhoods, resulting in a standardized mortality ratio (SMR) of 4.3 (Table 1). This indicates that child mortality in the most deprived neighborhoods was 330% higher than expected if fatalities were randomly distributed throughout the study area. In contrast, the least deprived neighborhoods had an SMR of 0.16, indicating significantly fewer child deaths than expected. These neighborhoods were also characterized by significantly higher levels of racial/ethnic homogeneity, concentrated disadvantage, and residential instability. Conversely, the most deprived neighborhoods had lower concentrations of immigrants and spent a smaller proportion of their income on housing and transportation costs.

**Table 1:** Descriptive characteristics of key study variables.

Characteristic	Least Deprived 10% of Neighborhoods (Q1, N = 132)^1^	Most Deprived 10% of Neighborhoods (Q10, N = 132)^1^	Diff.^2^	*p*-value^2^
Economic Hardship & Inequality	74.4 (2)	142.0 (10)	−68	<0.001
Observed Child Death Rates	0.11 (0.34)	1.59 (1.53)	−1.5	<0.001
Expected Child Death Rates	0.70 (0.49, 0.84)	0.41 (0.26, 0.59)	0.23	<0.001
Standardized Incidence Ratio (SIR)	0.16 (0.51)	4.30 (6.20)	−4.1	<0.001
Residential Racial Segregation (HHI)	0.30 (0.14)	0.14 (0.14)	0.16	<0.001
Concentrated Disadvantage	−0.95 (0.30)	1.69 (0.74)	−2.6	<0.001
Residential Instability	−1.35 (0.45)	1.26 (0.52)	−2.6	<0.001
Immigrant Concentration	0.04 (0.04)	0.02 (0.03)	0.02	<0.001

^1^Mean (SD); Median (IQR).

^2^Welch Two Sample t-test.

^3CI^ = Confidence Interval.

**Fig 2:**
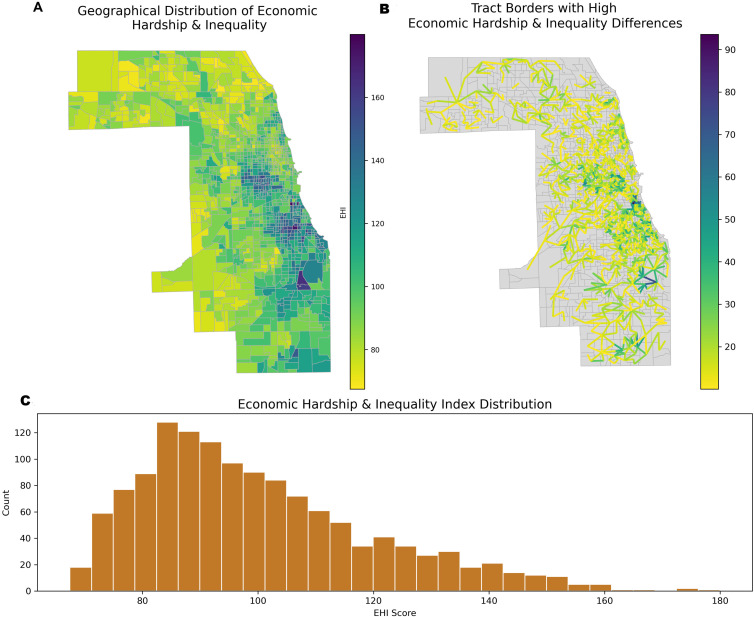
Distribution of Economic Hardship and Inequality. This figure shows the spatial pattern of the Economic Hardship and Inequality (EHI) Index across Chicago census tracts (A: top left), the locations of tract borders with high EHI differences (B: top right), and the overall distribution of EHI scores (C: bottom panel). Panel A (left) reveals a strong clustering of EHI, with the most disadvantaged tracts mainly on Chicago’s South and West Sides. In contrast, areas with lower EHI scores, indicating greater economic advantage, are visible in the northern and southwest parts of the county. Panel B (right) highlights social frontiers, defined as the boundary lines between neighboring tracts that show differences in EHI. These lines are drawn between tract centers, and the color indicates the size of the EHI difference. High-magnitude frontiers are mostly found along boundaries that separate the most disadvantaged neighborhoods from their wealthier neighbors. These areas mark points of sharp structural inequality and sudden changes in hardship over small distances. Panel C presents a histogram of EHI scores across all tracts, showing a right-skewed distribution with most tracts clustered around moderate hardship levels (85–110) and a smaller number of tracts with very high scores (above 140), highlighting the concentration of severe disadvantage in certain areas.

Moran’s I = 0.76 (**p* *< .001) for the EHI indicates strong positive spatial autocorrelation, meaning that areas with similar levels of economic hardship are geographically clustered. Fig 2A illustrates the geographic distribution of EHI across census tracts in Cook County, visually showing how EHI values are clustered across the region. Fig 2B shows that many areas are characterized by substantial differences in EHI across neighboring areas, some of which appear stark. The histogram in Fig 2C displays a right-skewed distribution in EHI, indicating that most neighborhoods’ EHI levels are clustered around the mean (100), with fewer neighborhoods having levels above the mean, and very few having extremely high levels of EHI (>140).

**Fig 3:**
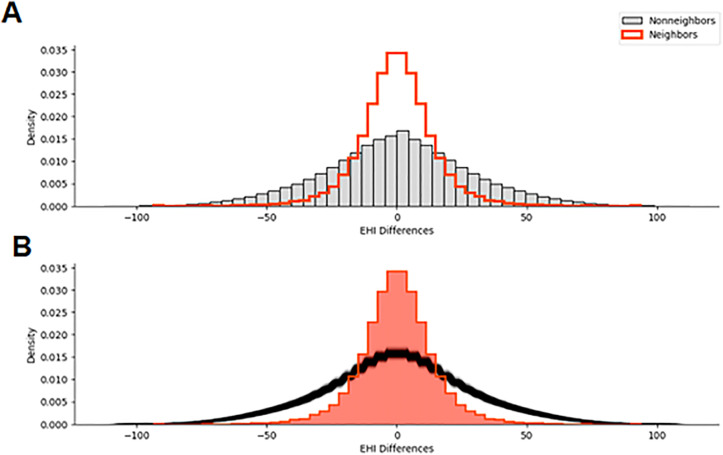
Neighborhood Differences in Economic Hardship and Inequality. Distribution of differences in the Economic Hardship and Inequality (EHI) across pairs of neighboring census tracts (A) and the randomization process used to detect significant differences **(B)**. The peak of the histogram centered around zero represents no difference in EHI between tracts, meaning that two census tracts have the same level of EHI.

Fig 3 shows the distribution of differences in the EHI across all pairs of census tracts. Panel A compares the observed EHI differences among neighboring tracts (in red) to those among non-neighboring tracts (in gray). The distribution for neighboring tracts is more narrowly concentrated around zero, indicating that adjacent tracts tend to have more similar EHI levels, a pattern consistent with positive spatial autocorrelation. In contrast, the broader distribution for non-neighbors reflects larger variability in EHI between tracts that are not spatially adjacent. Panel B shows the results of a Monte Carlo randomization procedure [78], where tract boundaries were shuffled to generate a null distribution of EHI differences under spatial randomness. The observed distribution for neighboring tracts (shaded red) is significantly more concentrated than the randomized distribution (black), confirming that the similarity in EHI among neighbors is not due to chance. This provides evidence of a localized spatial structure, where EHI values cluster geographically and spatial discontinuities can emerge between adjacent tracts. Identifying these local boundaries using spatial dissimilarity modeling was the goal of the subsequent analysis.

To do so, we implemented three model specifications: (1) an intercept-only model with EHI-based boundaries; (2) a model incorporating social disorganization variables; and (3) a full model including spatially lagged covariates to capture contextual effects from surrounding tracts. Table 2 summarizes the findings from each model specification, including the log-relative risk estimates for covariates (e.g., concentrated disadvantage), the intercept term, the variance of the spatial random effects (τ²), and Geweke’s diagnostic for model convergence. Although the model estimates a spatially structured log-risk surface ($\log\left(R_{k}\right)$), those values are not shown in Table 2. Rather, our primary focus is on the estimated effect of living near a social frontier, which is captured by the term labeled “Boundary: EHI” in Table 2. The posterior distribution of the dissimilarity coefficient α is compared against a threshold value $\alpha_{\min}$; values above this threshold indicate that the dissimilarity metric (e.g., EHI decile difference) is effective at identifying true boundaries in the risk surface [71,72]. Here, $\alpha_{\min}\;$refers to the threshold value below which dissimilarities (in EHI) are not treated as meaningful boundaries in the spatial random effects surface. In all three models, the posterior mean and 95% credible interval for α lie above $\alpha_{\min}=0.077$, indicating strong evidence that EHI boundaries are meaningful sites of discontinuity in child mortality risk.

**Table 2:** Results from the Spatial Discontinuity Models.

Parameter	Model 1	Model 2	Model 3
Ln Relative Risk (Intercept)	–0.655 (–0.787, –0.535)	–0.398 (–0.695, –0.140)	–0.429 (–3.059, 1.927)
τ² (Variance of RE)	1.664 (1.231, 2.092)	0.962 (0.528, 1.447)	0.584 (0.327, 0.899)
HHI	—	–0.062 (–0.579, 0.582)	–0.102 (–1.029, 0.574)
Concentrated disadvantage	—	0.244 (0.113, 0.362)	–0.007 (–0.173, 0.141)
Residential instability	—	0.291 (0.149, 0.427)	0.061 (–0.111, 0.226)
Immigrant concentration	—	–2.233 (–3.590, –0.946)	–1.872 (–4.240, 0.509)
HHI lag	—	—	0.375 (–0.931, 1.721)
Concentrated disadvantage lag	—	—	0.580 (0.300, 0.833)
Residential instability lag	—	—	–0.186 (–0.472, 0.108)
Immigrant concentration lag	—	—	–1.919 (–5.249, 0.378)
Boundary: EHI	0.200 (0.174, 0.227)	0.188 (0.038, 0.229)	0.193 (0.091, 0.228)
$\alpha_{\min}$	0.077	0.077	0.077
Step changes identified	413	413	413
DIC	2280.42	2279.60	2260.11
Effective parameters (p.d.)	201.60	143.10	119.60
LMPL	–1145.26	–1145.18	–1136.26

***Table notes.*** Ln = log. EHI = Economic Hardship and Inequality; The boundary EHI quantifies the effect of living in a ‘social frontier’ on the relative risk of child fatality in a given census tract. HHI = Hirschman-Herfindahl Index is a measure of racial/ethnic segregation in a given census tract. The lag variables capture the influence of surrounding regions’ on that measure on the child death outcomes in the focal region. $\alpha_{\min}$ is the posterior threshold for determining whether dissimilarity values identify boundaries in the risk surface. Step changes reflect the number of tract borders identified as spatial discontinuities. DIC = Deviance Information Criterion; LMPL = Log Marginal Pseudo-Likelihood; p.d. = number of effective parameters; RE = random effects.

In Model 1, which includes only the intercept and boundary variable, the effect of living on a boundary is associated with a 22.2% increase in relative risk of child mortality $\left(\exp^{0.2002}=1.2216\right)$. The posterior mean and 95% credible interval of the regression parameter α lie completely above the threshold ($\alpha_{\min}$), indicating that the EHI dissimilarity metric identifies statistically significant boundaries. In Model 2, we added four indicators of social disorganization—concentrated disadvantage, HHI, immigrant concentration, and residential instability. Controlling for these factors, concentrated disadvantage and residential instability remained significantly associated with increased risk, while immigrant concentration was associated with a protective effect. The effect of the boundary variable persisted, although its magnitude slightly declined.

Consistent with prior sociological and geographic research [49,79], we included spatially lagged versions of all four covariates in Model 3. Model 3 achieved the best overall fit (DIC = 2260.11, LMPL = –1136.26), outperforming Models 1 and 2 in both parsimony and explanatory power. Notably, the EHI boundary effect remained statistically significant across all models, supporting the conclusion that sharp economic disparities across neighborhood boundaries play a significant and independent role in shaping child mortality risk.

**Fig 4:**
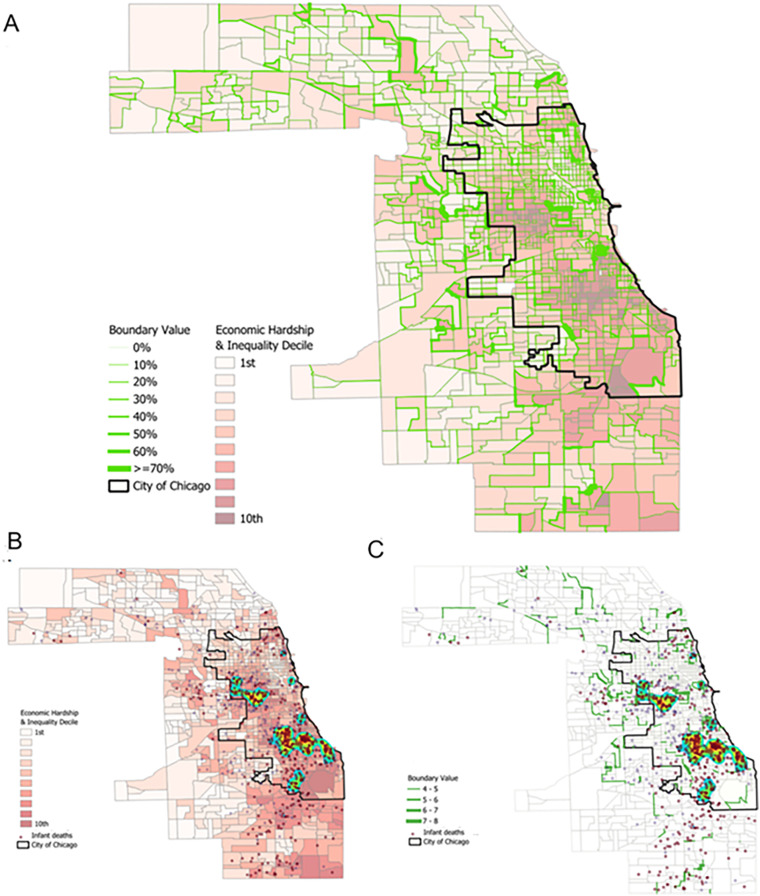
Spatial Frontiers and Child Mortality. This figure presents the spatial distribution of economic hardship and inequality (EHI) alongside social frontiers and child deaths across Cook County census tracts. Panel A: Tracts are shaded by EHI decile (1st = least hardship, 10th = most hardship). Overlaid green lines indicate the probability that a boundary represents a significant spatial discontinuity in child mortality risk, with line thickness corresponding to the magnitude of the posterior boundary effect. Darker and thicker green lines represent stronger evidence of a risk discontinuity across that boundary. Black lines delineate the municipal boundary of the City of Chicago. Panel B: Infant deaths are plotted as dots over the EHI decile map to show spatial concentration. Darker red shading reflects greater hardship. Infant fatalities cluster heavily in neighborhoods with high EHI on Chicago’s South and West Sides. Panel C: This panel isolates only the most extreme boundaries—those with EHI differences of ≥ 4 deciles (green lines)—and overlays infant death locations. The lines mark transitions between high- and low-hardship neighborhoods and are visually associated with elevated concentrations of infant deaths along neighborhood divides.

Across all models, posterior medians, 95% credible intervals (crI), and minimum threshold values ($\alpha_{\min}$=0.077), are reported for the economic inequality variable, confirming that the EHI dissimilarity metric consistently detects boundaries in the risk surface. In Fig 4A, the 413 boundaries are represented as solid green lines, with line thickness corresponding to higher probabilities of child mortality risk. The most significant boundaries are located at the divisions between regions in the Northeast and Midwest sections of the county, with the most prominent boundary being the one between the central-western and northeastern parts of Chicago. The boundaries identified in the analysis represent 10.6% of all borders between census tracts in the study region, indicating that a relatively small but meaningful portion of the census tract boundaries are associated with sharp contrasts in economic inequality (Fig 4B). Even after controlling for multiple social disorganization factors and their spatially lagged effects, the influence of economic inequality boundaries on child mortality remains significant. These findings highlight the role of stark EHI contrasts at neighborhood boundaries in increasing the risk of child mortality, independent of internal neighborhood characteristics and broader spatial processes (Fig 4C) [49].

## Discussion

Rather than treating neighborhood borders as static political boundaries, this study aimed to identify *spatial discontinuities* in economic inequality —defined as sharp differences in socioeconomic conditions across neighborhood borders —and their impact on the risk of child mortality. This approach is particularly salient in places like Chicago, where decades of de jure segregation have shaped persistent racial and economic inequality [80,81]. While extensive research links neighborhood deprivation to child health outcomes [82], few studies have examined whether relative deprivation across adjacent neighborhoods adds unique explanatory power beyond broader socioeconomic gradients [55]. Our findings provide the first county-wide evidence that census tract boundaries characterized by sharp differences in EHI, which we refer to as *social frontiers,* are associated with a 22% higher relative risk of child mortality, net of traditional measures of social disorganization, including racial segregation, concentrated disadvantage, residential mobility, and immigrant concentration. Descriptive patterns further support the role of inequality in shaping child health. The SIR of child deaths was 330% higher in the most deprived 10% of neighborhoods than in the least deprived, consistent with prior evidence of socioeconomic gradients in child well-being [51]. The results of this study align with previous research, which demonstrates how social and spatial factors shape local opportunities, resulting in extreme differences in socioeconomic status and affecting the health outcomes of children living in areas of social deprivation [83].

The spatial distribution of EHI in Cook County neighborhoods demonstrated that areas of extreme advantage are concentrated in some regions of the city. Further, neighborhoods that are closer together are characterized by smaller differences in EHI compared to neighborhoods that are farther away. Despite EHI’s strong spatial clustering, we found substantially less variability in the least deprived 10% of neighborhoods compared to the most deprived 10%. We also observed localized deviations from the predominant pattern where extreme differences in EHI are characterized by latent discontinuities in the risk surface. These findings suggest that the geographic concentration of advantage contributes more to inequality in child mortality than the concentration of EHI, given the increased risk of child mortality in areas categorized as social frontiers.

To reconcile the broader concentration effect with the local frontier effect, we emphasize that both contribute to child mortality risk, but in different ways. While overall neighborhood disadvantage is a strong predictor of poor health outcomes, our results suggest that local discontinuities—where advantaged and disadvantaged neighborhoods intersect—exert additional risk beyond what would be expected from deprivation alone. This aligns with Kramer’s concept that spatial boundaries can “orient and abet other inequalities by reifying differences between otherwise similar urban spaces” [45]. Similarly, the number of social frontiers uncovered in this study provides evidence of relative resource deprivation across Cook County [56]. The study found that neighborhoods bordered by areas with higher levels of concentrated disadvantage and residential instability faced increased risks of child mortality, suggesting a spatial spillover effect. In these areas, relative resource deprivation may affect a family’s ability to meet its basic needs with the resources available in the community [57]. As a result, neighborhoods with elevated levels of disadvantage and instability not only have higher mortality rates within their boundaries but also contribute to higher mortality rates in neighboring areas. These stark contrasts in EHI reflect economic disparities and have direct consequences for child health, illustrating how location can impact access to resources and differentially shape opportunities across socioeconomic divides.

### Implications for policy and practice

The results of this study hold several public policy implications relevant to child health outcomes. Considering our results, policies aimed at reducing economic inequality rather than poverty or increasing income can play a vital role in mitigating child mortality risks. For example, cash transfer programs that reduce poverty, improve economic autonomy, and enhance child nutrition and health service utilization are associated with significant reductions in mortality among children under five years of age and women [84]. However, while such programs reduce poverty, they have been shown to have a more limited impact in reducing inequality [85]. On the other hand, mixed-income housing developments may effectively address inequality with other interventions that reduce prejudice and intergroup competition, facilitate civic participation, and support social well-being in built environments [86]. There are other effective ways to address economic inequality by focusing on neighborhood transformation through place-based initiatives [87], such as the Dudley Street Initiatives Promise Neighborhoods Program in Boston [88]. Place-based strategies such as this deliver comprehensive social services, healthcare, education, and child welfare, and can help families living in more deprived areas receive the same level of support as those in more affluent areas. By addressing neighborhood inequalities directly, these programs provide children with spatial access to quality education, healthcare providers, and safer environments, thereby indirectly reducing the risk of child mortality [89]. By focusing on EHI, these interventions not only reduce child mortality, but they also foster long-term resilience in the most disadvantaged neighborhoods.

Social frontiers should be public health priority areas where interventions are focused on bridging the gap in EHI between affluent and deprived areas through infrastructure and community development initiatives. These initiatives include affordable community centers and healthcare facilities that offer multifaceted services conditioned by local needs. To address spatial discontinuities in EHI, interventions to reduce child mortality risk should focus on localized opportunity structures rather than administratively defined areas [90]. This includes services targeted to areas of relative resource deprivation, i.e., “social frontiers” that serve both sides of the divide but are specifically designed to reduce the stark inequalities. Implementing mobile health clinics or social services that specifically target neighborhoods at social frontiers can improve access to healthcare in disadvantaged areas. Targeting areas of concentrated disadvantage directly confronts the socioeconomic divides that contribute to higher child mortality rates.

### Limitations

This study highlights the crucial role of neighborhood environments in shaping health and socioeconomic disparities. Our conceptual and methodological approach emphasizes the significance of spatial factors in shaping policies aimed at enhancing child well-being, highlighting neighborhood boundaries and economic disparities as crucial elements in reducing child mortality, particularly in urban areas. Nevertheless, our study is not without limitations. First, the use of area-level data, such as census tracts, may obscure finer-scale variations within neighborhoods, limiting the ability to capture small-scale dynamics that influence child mortality. Additionally, the model assumes linear relationships between socioeconomic inequality (EHI) and child mortality, which may not fully capture more complex interactions. There is also the potential for unmeasured confounding factors that we did not consider that are known to influence both socioeconomic inequality and child mortality rates. In particular, future studies would benefit from incorporating additional covariates, such as availability of health care, natural environmental exposures, and aspects of the built environment to more fully capture the contextual factors influencing child mortality risk across neighborhood boundaries. Additionally, the use of longitudinal designs would allow researchers to explore how temporal shocks, such as the COVID-19 pandemic, interact with spatial risks over time. Additionally, moderate levels of multicollinearity in our spatially lagged model may contribute to coefficient suppression or unreliability, warranting caution when interpreting the results to avoid overinterpreting the direction or magnitude of the coefficients. The cross-sectional nature of the data limits the ability to account for temporal dynamics, and the findings may not generalize beyond Cook County, Illinois. Although our results were robust to alternative specifications for the EHI dissimilarity metric, using other indicators of socioeconomic deprivation may result in different results. These limitations should be considered when interpreting the findings, and future research would benefit from replicating our results to provide more insight into the impact of broadly defined inequality on child mortality.

## Conclusion

In summary, spatial spillover in child mortality across neighborhoods is a complex phenomenon influenced by many factors. Understanding this can help develop targeted interventions to address these disparities and improve child health outcomes [1]. The study underscores the importance of targeted interventions considering different neighborhoods’ specific characteristics that reduce economic inequality, including secure housing and accessibility to transportation and jobs, paying particular attention to how it plays out over geographic space [3].
